# Evidence-based public health: not only whether it works, but how it can be made to work practicably at scale

**DOI:** 10.9745/GHSP-D-14-00066

**Published:** 2014-08-31

**Authors:** James D Shelton

**Affiliations:** a Global Health: Science and Practice, Editor-in-Chief.

## Abstract

Because public health must operate at scale in widely diverse, complex situations, randomized controlled trials (RCTs) have limited utility for public health. Other methodologies are needed. A key conceptual backbone is a detailed “theory of change” to apply appropriate evidence for each operational component. Synthesizing patterns of findings across multiple methodologies provides key insights. Programs operating successfully across a variety of settings can provide some of the best evidence. Challenges include judging the quality of such evidence and assisting programs to apply it. WHO and others should shift emphasis from RCTs to more relevant evidence when assessing public health issues.

## WHAT MAKES PUBLIC HEALTH DIFFERENT FROM INDIVIDUAL MEDICAL CARE? SCALE, SITUATION VARIABILITY, AND INTERVENTION COMPLEXITY

Evidence-based medicine (EBM) has greatly advanced the scientific validity, and presumably the effectiveness, of medical practice.^1^ The hallmark of EBM is the randomized controlled trial (RCT) with its potentially strong “internal validity” to answer precise questions under narrow conditions—generally whether and how well an intervention such as a drug works for individuals. Indeed, the Cochrane Review process—the mainstay of EBM—places RCTs on a pedestal above all other forms of evidence. There is a strong temptation to apply EBM methods and standards reflexively to public health. The World Health Organization (WHO), for example, relies heavily on the GRADE (grading of recommendations, assessment, development, and evaluation) system, which gives paramount importance to RCTs, to develop recommendations for public health issues.^2^

But public health must operate at large scale, addressing the needs of large populations across clinical, behavior, and structural platforms, and necessarily entails crucial operational issues, variability, and complexity as well as consideration of resource requirements and sustainability. Thus, because situations can vary so widely, “external validity” or generalizability of evidence to other situations is absolutely crucial for public health applications.

Yet external validity is a severe weakness of the RCT methodology for public health decision-making. We need to know not just whether something works under narrowly prescribed circumstances but also how, when, and why it can work for broad application. Actually, the distinction between these 2 arenas is already recognized to some extent in standard terminology. We use the term “efficacy” to mean how well an intervention works under the best circumstances, typically based on RCT evidence, and “effectiveness” to mean actual results, especially at scale, which are usually attenuated under real-world conditions.

Public health decision-making requires knowledge of not just whether something works under particular circumstances but also how, when, and why for broad application.

But real-world complexity extends even farther. Many of the world's leading health agencies are committed to ending preventable infant and maternal death.^3^ This ambitious objective will require a considerable range of interventions, such as immunization, antibiotics for pneumonia, and promotion of exclusive breastfeeding. However, since no single intervention will be sufficient, the fundamental challenge is maximizing the *collective* effectiveness of the optimal set of interventions. That gives rise to a higher level of complex questions: What is the best set of interventions for particular settings? How should the interventions be organized and delivered within existing systems? What will it take to execute them on an ongoing, sustainable basis? With what effort and cost? What strategies are needed to best reach those most in need?

The deeper real-world understanding we need in public health actually serves 2 related purposes. The first is to **assess** whether and under what circumstances to recommend a particular public health approach. The second is to help guide program managers to **apply** these approaches in their particular settings. To address such questions, evidence-based public health (EBPH) must go well beyond RCTs to include other valid methodologies to arrive at optimal public health programming. More challenging, but more interesting.

## LIMITATIONS OF RCTS FOR PUBLIC HEALTH

RCT methodology entails a host of challenges, including burdensome cost and time requirements. Sometimes randomization is not possible for ethical or logistical reasons. But crucially, RCTs are necessarily “**reductionist**,” which limits their utility for complex public health issues. With laser-like precision, they typically zero in on very specific issues in constrained time and place. Because human biology is pretty consistent across time and place, that often works quite well for medical questions about individuals. But major variability can occur even for biologic questions. For example, trivalent polio virus vaccine is quite efficacious in the developed world. However, in Northern India vaccine efficacy was only 9% per dose.^4^ Possible explanations offered include co-infection, malnutrition, genetic differences, size of viral inoculum, and enteropathy. Moreover, since RCTs are “controlled,” they are typically carried out under optimal and rather **artificial** conditions, which are frequently difficult to transfer to practical real-world conditions.

Challenges to conducting RCTs are many, only beginning with high cost and time requirements.

### Complex Interventions in Complex Environments

Consider the challenges of designing and implementing public health programs at scale. Contexts vary widely, especially related to culture, social structure, health systems, resources, economics, politics, and the physical environment. Furthermore, services can be delivered through a range of modalities, each with a multitude of permutations of how the services can be organized. Trying to apply the laser-like RCT approach is akin to trying to light up a football stadium with a slowly moving laser pointer—very precise, rigorous, and artificially intense but not very illuminating.

Trying to apply the laser-like RCT approach is akin to trying to light up a football field with a slowly moving laser pointer—very precise, rigorous, and artificially intense but not very illuminating.

### Examples of Problematic Application of RCTs to Public Health

The deference for RCTs in EBM has spilled over to public health, for example, via “cluster” randomized trials that randomize population groups rather than individuals. But typically so many variables affect the result, and the trials are often conducted under rather artificial optimal conditions, that generalizing to the variable range of real-world situations can be tenuous at best. Moreover, research culture tends to focus on the “whether” questions, driving study designs, data collection, and what scientific journals like to report. Alas, the crucial questions of “how” and “under what circumstances” typically get short shrift. Some examples:

- **Variability and failure to assess the causal pathway fully.** It is believed concurrent infection with certain sexually transmitted infections (STIs) may facilitate HIV transmission. A large and expensive cluster randomized trial in Mwanza, Tanzania, found that when STIs were treated, HIV transmission decreased. However, several other population-level RCTs failed to show such a reduction.^5^ Unfortunately, while measuring HIV incidence, the Mwanza study failed to adequately document whether the crucial intervening variable—prevalent STIs—was actually reduced. Subsequent commentators have speculated that the different study outcomes might have resulted from differences in the existing burden of STIs and from the intense, active phase of the HIV epidemic in Mwanza at the time.^5^ Regrettably, the role for STI treatment in HIV prevention remains rather unresolved.
- **Overgeneralization and lack of process detail.** A cluster randomized trial involving peer mentors with HIV infection to promote wellness behavior among pregnant South African women living with HIV (WLH) drew this unqualified conclusion: “WLH benefit by support from HIV-positive peer mentors …” Actually, the study was small and localized to one area of South Africa; it found significant and mostly modest change in only 4 of 19 behaviors (mostly related to improvements in exclusive breastfeeding); and it provided little intervention detail and no qualitative evidence about the thinking and motivations of the WLH.^6^ Moreover, participation in the study was partial and follow-up rates rather incomplete.

In fairness, some cluster trials do include complementary methodologies beyond simply whether the intervention worked or not. Notably, the very large Project Accept study, which is assessing whether widespread HIV testing plus community engagement reduces risky behavior, includes a major ideational component assessing attitudes and motivation.^7^

## THE CRUCIAL DETAILS: CONSTRUCTING A CAUSAL PATHWAY OR THEORY OF CHANGE

Properly implementing a public health initiative involves engaging a plethora of issues that can include constellation of interventions, staffing, deployment, job functions, competence, motivation, compensation, program policies, organization of work, standards and guidelines, job aids, quality assurance, supply chain, physical infrastructure, budget, cost recovery, demand creation, healthy behavior promotion, public support, supervision, change management, epidemiologic surveillance, and service data collection and use, to name a few—all customized to the dynamic local context.

Real-world managers typically address these challenges through intuition, trial and error, and experience. That is actually not a bad starting point. After all, it is how our species survived through millennia in a complex environment. However, this intuitive process can be improved by systematically laying out a posited causal pathway, sometimes called a “theory of change,” of the steps and components that need to happen to get the desired results. The task then becomes identifying the best evidence, both internal and external, on what helps each component, as well as the whole, to function better.

## WHAT SHOULD WE USE FOR EBPH EVIDENCE?

EBPH approaches have much in common with management science. Both use experiment-like tests of effectiveness but must rely heavily on evidence that is observational, experiential, or essentially systematic trial and error. Validity often derives from whether things “work” in a particular environment. Broader applicability emerges when consistent patterns of findings or collective “lessons learned” materialize. Some examples:

Patterns of findings or collective “lessons learned” that are consistent across multiple settings are especially valuable for EBPH.

- **Successful implementation/positive deviance.** One major way of addressing the crucial issues of scale and complexity is examining what actually works (or not) at scale, and then parsing the details. Such a “case study” or positive deviance approach is a backbone of business schools. This approach can also be comparative. For example, the management classic *Built to Last: Successful Habits of Visionary Companies* compares the attributes of highly successful companies matched with less successful ones.^8^ When a repeated pattern of success is seen across many different situations, it provides confidence in the general approach. Accordingly, a family planning nongovernmental organization, Marie Stopes, successfully provided more than 700,000 contraceptive implants in 2012 across a wide variety of countries in sub-Saharan Africa. They describe their 3 service delivery modalities, along with operational details including provider training, client outreach, robust supply chains, and quality assurance measures. A generalizable concept or best practice that emerges is the “dedicated” provider for such labor-intensive contraceptive methods.^9^
- **Systematic trials and program tests.** This category includes a wide variety of methodologies, ranging from randomized trials and quasi-experimental designs to demonstration projects. Such investigations (including RCTs) should provide extensive detail on what did and did not work, as well as how. In the 1970s, studies in many settings found that community-based provision of family planning was acceptable to a substantial proportion of couples, making expanding contraceptive access a major pillar of family planning programming.^10^ Likewise, a recent analysis of 12 successful community-based child survival projects found intensive outreach to caregivers and community leaders was a crucial common element.^11^
- **Performance improvement.** In this approach, with its roots in management science, managers, typically along with staff, assess critical strengths and weaknesses in programs using a variety of analytical tools. They formulate solutions, test them, and measure whether and how performance improves. Generalized knowledge can arise when patterns of solutions emerge, common across multiple program experiences. Thus for male circumcision, a variety of incremental improvements in mobile service delivery have been identified, including client preparation outside the facility, use of a forceps-guided surgical procedure, reorganized bed use, task shifting, and task sharing, that have resulted in substantial increases in efficiency with good quality.^12^
- **Simple measurement across the causal pathway.** A wide variety of data can be useful to assess and guide implementation such as: routine service provision data, qualitative data on client or provider perspectives, facility assessments, supply chain monitoring data, epidemiologic surveillance, fixed-interview surveys such as the Demographic and Health Surveys (DHS), costing data, national health account data to assess health spending, mapping data on location of facilities and transport networks, and other key data such as air quality and food, alcohol, and tobacco consumption. In addition to using such data for direct program assessment, useful general patterns can emerge. For example, a worldwide analysis based on DHS data revealed that a substantial proportion of people access the private sector for key child health services in many developing countries, arguing for more programmatic effort to engage private-sector providers.^13^
- **Additional epidemiologic methods.** These include cohort and case-control studies to help assess factors predicting health, disease, and adverse outcomes, as well as phylogenetic studies to assess patterns of disease transmission.
- **Modeling.** While modeling doesn't actually generate new data, this exploration of the *implications* of data can provide insights into whether, when, and how interventions may work. For example, antiretroviral drugs (ARVs) reduce HIV transmission, but their population-level potential to abate the HIV epidemic is unclear.^14^ Credible modeling indicates that investing in treating those already infected, especially with more advanced disease, is more cost-effective than providing ARVs to those uninfected but at risk of infection.^15^ But beware. The greater the situational complexity, the more variability can arise from the assumptions and model mechanics. Modeling can definitely be misleading.
- **Human functionality, culture, and biology.** This approach, which includes both experimental and observational methods, is somewhat reductionist and far-ranging. It can comprise task analysis issues, such as how many clients a provider can effectively see daily, how many tasks a community health worker can effectively provide, and what training approaches result in competence; how medical culture influences programs; how social networks influence behavior; and what neuroimaging changes correlate with approval or disapproval when individuals see an anti-smoking ad.
- **Evaluation.** Summative evaluations that assess program effectiveness can be designed in advance as well as conducted post hoc. They can make use of a variety of methods as described above. Here also, it is important not only to assess whether something worked or how well it worked but also to uncover the details of the many factors that caused it to work or not. Probably the paradigm of ideal evaluation is the “Realist Review.”^16^ This approach “aimed at discerning what works for whom, in what circumstances, in what respects, and how” is a deep-dive approach to evaluation,^16^ beginning with a detailed theory or causal chain on how an intervention is intended to produce impact and then populating the series with available empiric evidence.

## QUALITY OF EBPH EVIDENCE

There are well-established criteria for assessing the quality of RCTs. However, addressing quality of evidence in the complex and variable terrain of public health, with its diverse and more complex questions (when, how, how cost-effective, and how sustainable) and with its heterogeneous forms of evidence, is far less cut-and-dried. Nevertheless, quality criteria for methodologies such as qualitative approaches do exist. Pawson lays out key principles for quality of evidence in his seminal paper, “Assessing the quality of evidence in evidence-based policy: why, how and when?”^17^ Ultimately, in addition to specific criteria for particular methodologies, he judges quality of studies based on how well they contribute to or triangulate with other evidence, in coherent and credible explanatory patterns. We need to build on such concepts to further refine and assess the quality of public health evidence.

A starting point for assessing the quality of EBPH evidence is how well the studies contribute to other evidence.

## SYNTHESIS FOR APPLICATION, THE ULTIMATE CHALLENGE FOR EBPH

A key value-added of EBPH is identifying and synthesizing patterns of findings across multiple experiences, less than perfect though they may be, in enough detail to meaningfully inform similar efforts across a variety of situations. Simple examples I personally have observed include:

- Referrals from one place in the health system to another risk a high loss to follow up.
- Success in programs, including scaling up, often depends on finding and cultivating committed and capable “champions.”
- Important population-level behavior change, such as reducing tobacco use, most often results not from any one single campaign or intervention but from a sustained combination of interventions, including structural interventions such as increasing taxation, individual persuasion, and changing social norms.

Another example of synthesis is a systematic review of strategies to increase health services in mountainous locations. It found benefit from: task shifting, strengthened roles of community health workers, mobile teams, and inclusive structured planning forums.^18^ A major agenda for EBPH is identifying such common patterns and helping program managers adapt and apply that knowledge.

Synthesizing common patterns across multiple methodologies and helping program managers apply that knowledge are key goals for EBPH.

## CONCLUSION

To achieve ambitious global health goals, such as ending preventable child and maternal mortality, we need evidence on the “how and when” of implementation at scale, in the face of vast real-world complexity and situational variability. Evidence arising within a specific program can help with better implementation in that setting. But beyond locally relevant learning, a major objective is identifying systematic patterns for wider application. Triangulating and otherwise bringing together evidence arising from different methodologies with sufficient detail to illuminate causal relationships is essential to applying such knowledge to real-world public health problems across diverse situations. When assessing public health evidence, WHO and others should move beyond predominant reliance on RCT evidence.

Some may question the rigor of these approaches. But we are not advancing mere anecdote. Rather, our mandate is an even greater and more difficult standard of rigor: of investigation, observation, accumulation, systemization, and appropriate application. Narrow internal rigor elegance is not an end in itself. The overriding virtue of EBPH is real-world relevance.

## References

[1] Rosenberg W Donald A Evidence based medicine: an approach to clinical problem-solving. BMJ. 1995;310(6987): 1122–1126 10.1136/bmj.310.6987.1122 7742682 PMC2549505

[2] World Health Organization (WHO). WHO handbook for guideline development. Geneva: WHO; 2012 Available from: http://apps.who.int/iris/bitstream/10665/75146/1/9789241548441_eng.pdf

[3] UNICEF. Committing to child survival: a promise renewed. Progress report 2013. New York: UNICEF; 2013 Available from: http://www.unicef.org/publications/files/APR_Progress_Report_2013_9_Sept_2013.pdf

[4] Grassly NC Fraser C Wenger J Deshpande JM Sutter RW Heymann DL New strategies for the elimination of polio from India. Science. 2006;314(5802): 1150–1153 10.1126/science.1130388 17110580

[5] Korenromp EL White RG Orroth KK Bakker R Kamali A Serwadda D Determinants of the impact of sexually transmitted infection: a synthesis of evidence from the Mwanza, Rakai, and Masaka intervention trials. J Infect Dis. 2005:191 (Suppl 1):S168–78 10.1086/425274 15627227

[6] Rotheram-Borus MJ Richter LM van Heerden A van Rooyen H Tomlinson M Harwood JM A cluster randomized controlled trial evaluating the efficacy of peer mentors to support South African women living with HIV and their infants. PLoS One. 2014;9(1):e84867 10.1371/journal.pone.0084867 24465444 PMC3898948

[7] Maman S van Rooyen H Stankard P Chingono A Muravha T Ntogwisangu J; NIMH Project Accept (HPTN 043) study team. NIMH Project Accept (HPTN 043): results from in-depth interviews with a longitudinal cohort of community members. PLoS ONE. 2014;9(1):e87091 10.1371/journal.pone.0087091 24489841 PMC3906114

[8] Collins J Porras JI Built to last: successful habits of visionary companies. New York: HarperCollins; 1994

[9] Duvall S Thurston S Weinberger M Nuccio O Fuchs-Montgomery N Scaling up delivery of contraceptive implants in sub-Saharan Africa: operational experiences of Marie Stopes International. Glob Health Sci Pract. 2014;2(1):72–92 10.9745/GHSP-D-13-00116 PMC4168608 25276564

[10] Prata N Vahidnia F Potts M Dries-Daffner I Revisiting community-based distribution programs: are they still needed? Contraception. 2005;72(6):402–407 10.1016/j.contraception.2005.06.059 16307960

[11] Ricca J Kureshy N LeBan K Prosnitz D Ryan L Community-based intervention packages facilitated by NGOs demonstrate plausible evidence for child mortality impact. Health Policy Plan. 2014;29(2):204–216 10.1093/heapol/czt005 23434515

[12] Mahler HR Kileo B Curran K Plotkin M Adamu T Hellar A Voluntary medical male circumcision: matching demand and supply with quality and efficiency in a high-volume campaign in Iringa Region, Tanzania. PLoS Med. 2011;8(11):e1001131 10.1371/journal.pmed.1001131 22140366 PMC3226544

[13] Hodgins S Pullum T Dougherty L Understanding where parents take their sick children and why it matters: a multi-country analysis. Glob Health Sci Pract. 2013;1(3):328–356 10.9745/GHSP-D-13-00023 PMC4168586 25276548

[14] Shelton JD HIV/AIDS. ARVs as HIV prevention: a tough road to wide impact. Science. 2011;334(6063):1645–1646 10.1126/science.1212353 22194560

[15] Cremin I Alsallaq R Dybul M Piot P Garnett G Hallett TB The new role of antiretrovirals in combination HIV prevention. AIDS. 2013;27(3):447–458 10.1097/QAD.0b013e32835ca2dd 23296196

[16] Pawson R Greenhalgh T Harvey G Walshe K Realist review - a new method of systematic review designed for complex policy interventions. J Health Serv Res Policy. 2005;10 Suppl 1:21–34 10.1258/1355819054308530 16053581

[17] Pawson R Assessing the quality of evidence in evidence-based quality: why, how and when? Swindon (UK): Economic and Social Research Council; [2007]. Available from: http://www.ccsr.ac.uk/methods/publications/Pawson.pdf

[18] Byrne A Hodge A Jimenez-Soto E Morgan A What works? Strategies to increase reproductive, maternal and child health in difficult to access mountainous locations: a systematic literature review. PLoS One. 2014;9(2):e87683 10.1371/journal.pone.0087683 24498353 PMC3912062

